# Tranilast Does Not Inhibit TRPV2

**DOI:** 10.3390/cells15010013

**Published:** 2025-12-21

**Authors:** Tabea C. Fricke, Nele Stein, Christine Herzog, Frank G. Echtermeyer, Andreas Leffler

**Affiliations:** 1 Department of Anesthesiology and Intensive Care Medicine, Hannover Medical School, Carl-Neuberg Strasse 1, 30625 Hannover, Germany; 2 PRACTIS Clinician Scientist Program, Dean’s Office for Academic Career Development, Hannover Medical School, Carl-Neuberg Strasse 1, 30625 Hannover, Germany

**Keywords:** TRPV2, tranilast, reactive oxygen species (ROS), ion channel modulation, channel pharmacology

## Abstract

**Highlights:**

**What are the main findings?**

- Tranilast does not directly block TRPV2 currents or calcium influx induced by established agonists.
- Tranilast reduces oxidation-induced TRPV2 modulation rather than direct channel inhibition.

**What are the implications of the main findings?**

- The long-held view of tranilast as a TRPV2 blocker should be reconsidered; its effects are indirect and redox-dependent.
- This work refines the pharmacological understanding of TRPV2 modulation and guides more accurate experimental and therapeutic use of tranilast in TRPV2 channel research.

**Abstract:**

Transient receptor potential vanilloid 2 (TRPV2) is a non-selective cation channel involved in diverse physiological and pathological processes. Tranilast has frequently been described and used as a rather specific inhibitor of TRPV2. However, the molecular basis of this inhibition was never been studied in detail. Here, we investigated whether tranilast indeed directly inhibits TRPV2. Rat TRPV2 was expressed in human embryonic kidney (HEK293) cells, and channel function was assessed using whole-cell electrophysiology and calcium imaging in response to established agonists. In parallel, we conducted phagocytosis assays in rat basophilic leukemia (RBL) cells, including a CRISPR/Cas9-generated TRPV2-knockout cell line. Tranilast up to 1 mM did not inhibit TRPV2-mediated currents or calcium influx induced by any agonist. However, when co-applied with the oxidant chloramine T, tranilast diminished oxidation-induced activation of TRPV2. This effect may indicate a general interference of tranilast with redox signaling. Accordingly, tranilast also reduced chloramine T-induced activation of TRPA1 as well as the development of non-inactivating currents of voltage-gated Na^+^ channels. Furthermore, tranilast decreased phagocytic activity in both wildtype and TRPV2-knockout RBL cells. However, the reduction was less pronounced in TRPV2-knockout cells. These findings demonstrate that tranilast does not directly inhibit TRPV2. Instead, tranilast seems to indirectly suppress channel activation by reducing reactive oxygen species (ROS). This refined understanding of how tranilast modulates TRPV2 has important implications for the interpretation of prior and future pharmacological studies targeting TRPV2.

## 1. Introduction

Transient receptor potential (TRP) channels form a superfamily of non-selective cation channels that act as cellular sensors for a broad range of chemical and physical stimuli. Among these, the vanilloid subfamily member TRPV2 is a Ca^2+^-permeable channel expressed in multiple tissues, including immune cells, cardiac myocytes, and sensory neurons [1,2,3]. TRPV2 contributes to processes such as innate immune responses, cardiac contractility, cancer cell migration, and inflammatory signaling [2,4,5,6,7]. Owing to its diverse physiological roles, TRPV2 has attracted interest as a potential therapeutic target in several pathological conditions.

Pharmacological tools are essential for probing TRP channel function and for exploring their therapeutic potential. While selective modulators are meanwhile well established for most TRP channels, the lack of TRPV2-selective pharmacological compounds leaves TRPV2 comparatively under-explored [8,9,10]. A limited number of unselective compounds have been used to inhibit TRPV2, including the synthetic drug tranilast [1,8,11,12,13,14,15,16,17]. Tranilast, a derivative of the amino acid tryptophan, is an anti-allergic and anti-inflammatory drug used clinically in Japan and Korea to treat asthma, allergic dermatoses, and to prevent hypertrophic scars and keloids [18]. It may also have broad therapeutic potential for treating several disease states, including fibrosis, proliferative disorders, cancer, cardiovascular conditions, and autoimmune conditions [18]. The link between tranilast and TRPV2 originated from research describing its ability to inhibit calcium entry in MCF-7 breast cancer cells, vascular smooth muscle cells and Chinese hamster ovary (CHO) cells [16,19,20]. In vascular smooth muscle cells, platelet-derived growth factor (PDGF) has been shown to evoke a biphasic increase in intracellular calcium, with a sustained phase dependent on extracellular calcium [19]. Tranilast abolished this response and inhibited PDGF-induced calcium influx and DNA synthesis in a concentration-dependent manner, without affecting upstream receptor phosphorylation or downstream signaling pathways [19]. Similar findings were later reported in MCF-7 breast cancer cells, where insulin-like growth factor 1 (IGF-1) induced oscillatory calcium elevations essential for cell-cycle progression [16]. Tranilast suppressed IGF-1–induced calcium entry and abolished these oscillations, suggesting that it acted on calcium-permeable channels rather than on receptor signaling [16]. Because TRPV2 was later identified as a calcium-permeable, growth factor–responsive cation channel [1,21,22], it was subsequently assumed that tranilast inhibits TRPV2. Despite the absence of direct experimental evidence confirming an actual TRPV2 involvement, tranilast has been designated a “selective TRPV2-blocker” [23,24]. The original studies reported substantial inhibition of calcium entry at concentrations around 60 µM, and near-complete blockade at approximately 100 µM tranilast. Nevertheless, later reports have commonly cited and used 75 µM tranilast as an effective concentration to inhibit TRPV2 [16,19,23]. Although more selective TRPV2-antagonists such as SET2 and IV2-1 are meanwhile available [25,26], tranilast is still commonly employed as a pharmacological tool to interfere with TRPV2 function. Recent reports have used tranilast to study the role of TRPV2 in macrophage activation and phagocytosis, the progression of dilated cardiomyopathy, and the prevention of right ventricular remodeling and arrhythmias in models of experimental pulmonary hypertension as well as pulmonary endothelial barrier recovery following reactive oxygen species (ROS)-induced permeability, fibrosis development and weight gain in models of non-alcoholic steatohepatitis (NASH), atrial fibrillation, amyloid-β–induced cognitive impairment as well as cancer [17,27,28,29,30,31,32,33,34]. In almost all these studies, the inhibitory effects of tranilast on calcium-dependent signaling were attributed to an inhibition of TRPV2. This interpretation extended beyond basic research and became the rationale for several clinical trials investigating tranilast in cardiac as well as oncological diseases [35,36,37,38,39,40]. The TRPV2-specificity as well as the mechanism of tranilast have been questioned in some studies [8,17,41]. Early work indicated that tranilast suppresses TRPV2-mediated currents evoked by mechanical stimulation, but that is ineffective when channel activation is induced by the chemical agonist 2-APB at high concentrations, e.g., suggesting stimulus-dependent effects [17,42]. Peralvarez-Marin and co-workers pointed out that although tranilast had been repeatedly described as a TRPV2-specific antagonist, this property had never been conclusively validated through detailed biophysical or structural studies [8]. Investigations in primary bronchial epithelial cells showed that both tranilast and a TRPV2-knockdown reduced ATP release in response to mechanical stress [41]. Yet, the authors acknowledged that the inhibitory mechanism of tranilast may not be confined to TRPV2 [41]. In addition to its reported actions on TRPV2, tranilast is known to modulate multiple cellular pathways, including inhibition of transforming growth factor-β signaling via suppression of SMAD4, interference with sepiapterin reductase activity and anti-proliferative effects [18,43,44,45]. These findings suggest that while tranilast can influence TRPV2-mediated cellular responses, its actions could as well be pleiotropic, and the extent to which it directly targets TRPV2 remains uncertain.

Yet, there is an evident lack of direct electrophysiological or biochemical evidence confirming the inhibition of TRPV2 by tranilast. The aim of this study was to clarify the mode of action of tranilast on TRPV2, providing a more accurate framework for interpreting studies that have used this compound as a TRPV2-selective pharmacological tool.

## 2. Materials and Methods

### 2.1. Chemicals

2-Aminoethoxydiphenyl borate (2-APB) was purchased from Tocris Bioscience (Bristol, UK) and prepared as a 100 mM stock solution in DMSO, stored at −20 °C. Cannabidiol (CBD) was obtained from Cayman Chemical Co. (Ann Arbor, MI, USA) and prepared as a 100 mM stock solution in DMSO, stored at 8 °C. Chloramine T was purchased from Sigma-Aldrich (St. Louis, MO, USA) and freshly prepared in external solution immediately prior to use. Tranilast was obtained from Biomol (Hamburg, Germany), dissolved in DMSO at 100 mM, and stored at −20 °C. Ruthenium red (RR) was purchased from Sigma-Aldrich (Taufkirchen, Germany) and prepared as a 10 mM stock solution in external solution, stored at 8 °C. For all experiments, RR was applied extracellularly at a final concentration of 10 µM together with CBD. RR was perfused as a control after establishing CBD-evoked TRPV2 currents and remained in the bath for at least 10–15 s. RR was used as a non-selective TRP channel blocker. All working concentrations were freshly prepared from stock solutions on the day of the experiment. Working concentrations of all compounds were freshly prepared from stock solutions on the day of the experiment.

### 2.2. Cell Culture

Stable rTRPV2, HEK 293t cells and hTRPA1 (PrecisION hTRPA1-HEK (RRID:CVCL_D1KL)) expressing HEK293 cells were maintained under standard conditions (37 °C, 5% CO_2_) in Dulbecco’s Modified Eagle’s Medium/Nutrient Mixture F12 (DMEM/F12; Gibco, Invitrogen, Darmstadt, Germany) supplemented with 10% fetal bovine serum (FBS; Biochrom, Berlin, Germany), 100 U/mL penicillin, 100 µg/mL streptomycin, 2 mM L-glutamine (Lonza, Verviers, Belgium), and G418 for selection in stable expressing cell lines. Stable rTRPV2 HEK293 cell lines were created as described previously [46]. HEK 293t cells were transfected with hTRPV2 and mTRPV2 plasmids using jetPEI (Polyplus-transfection SA, Illkirch, France). Dr Itaru Kojima (Gunma University) kindly gifted the cDNA of mTRPV2. ND7/23 cells (RRID: CVCL_4259) were cultured in DMEM/F12 (Gibco, Invitrogen, Darmstadt, Germany) supplemented with 10% FBS (Biochrom, Berlin, Germany), 100 U/mL penicillin, 100 µg/mL streptomycin, and 2 mM L-glutamine (Lonza, Verviers, Belgium). Rat basophilic leukemia cells (RBL-2H3; ATCC, CRL-2256; RBL-2H3 ADORA2A Gq (RRID: CVCL_KU64)) and TRPV2 knockout RBL cells (generated by a CRISPR/Cas9 induced premature stop codon in Exon 3 as described previously) were cultured in Eagle’s Minimum Essential Medium (EMEM; Bio&SELL, Feucht, Germany) containing L-glutamine and supplemented with 15% FBS (Biochrom, Berlin, Germany) [47]. All cell lines were maintained at 37 °C in a humidified incubator with 5% CO_2_.

### 2.3. Electrophysiology

Whole-cell or inside-out patch clamp recordings were performed on HEK 293 cells expressing TRPV2 or TRPA1, as well as on ND7/23 cells for experiments on voltage-gated Na^+^ channels. Currents were acquired using an EPC10 USB amplifier (HEKA Elektronik, Lambrecht, Germany), sampled at 2 (TRP channel) or 10 (Na^+^ channel) kHz, and filtered at 1 kHz. Patch pipettes were pulled from borosilicate glass capillaries (TW150F-3; World Precision Instruments, Sarasota, FL, USA) and had resistances of 2–5 MΩ. Whole-cell recordings were accepted only from cells with an access resistance below 10 MΩ. Series resistance compensation was only applied for experiments on Na^+^ channels. The extracellular recording solution for experiments on TRP channels contained (in mM): 140 NaCl, 5 KCl, 2 MgCl_2_, 5 EGTA, 10 HEPES, and 10 glucose, adjusted to pH 7.4 with NaOH. The pipette solution contained (in mM): 140 KCl, 2 MgCl_2_, 5 EGTA, and 10 HEPES, adjusted to pH 7.4 with KOH. Whole-cell experiments were performed on cells held at −60 mV. For inside-out multi-channel recordings performed at a holding potential of +60 mV, the solutions were reversed to 140 mM NaCl in the pipette and 140 KCl in the extracellular solution. For experiments on voltage-gated Na^+^ channels in ND7/23 cells, the extracellular recording solution contained (in mM): 140 NaCl, 2 MgCl_2_, 5 EGTA, 10 HEPES and 10 glucose adjusted to pH 7.4 with NaOH. The pipette solution contained (in mM): 140 CsF, 10 NaCl, 1 EGTA and 10 HEPES adjusted to pH 7.4 with CsOH. Cells were held at—120 mV and 20 ms long pulses up to 0 mV were applied at 0.1 Hz. All experiments were carried out at room temperature. Test solutions were applied using a gravity-driven multibarrel perfusion system composed of polytetrafluoroethylene and glass tubing. Data acquisition and analysis were carried out with Patchmaster v2x92 software (HEKA Elektronik) and Origin 8.5.1 (OriginLab, Northampton, MA, USA). For whole-cell recordings, current responses were quantified as current density (pA/pF). Current amplitudes were measured at the end of each drug application to ensure steady-state conditions. For inside-out recordings, responses were quantified as absolute current amplitudes (pA) determined at the end of each drug application.

### 2.4. Calcium Imaging

Cells were seeded on coverslips 24 h prior to measurements and loaded for 45 min with 4 μM Fura-2-AM in 0.02% pluronic. After washout, coverslips were mounted on an inverted microscope (Axio Observer D1; Zeiss, Jena, Germany). Fura-2 was excited at 340 and 380 nm using an HXP 120 light source (LEJ Lightning & Electronics, Jena, Germany) and a LEP filter wheel (Ludl Electronic Products Ltd., Hawthorne, NY, USA) with appropriate filter sets (Chroma Technology GmbH, Olching, Germany). Fluorescence images were acquired at 1 Hz with a CCD camera (CoolSNAP EZ; Photometrics, Puchheim, Germany) using exposure times of 10–20 ms. Data were recorded with VisiView 2.1.1 software (Visitron Systems GmbH, Puchheim, Germany).

The standard extracellular solution contained (in mM): 145 NaCl, 5 KCl, 1.25 CaCl_2_, 1 MgCl_2_, 10 glucose, and 10 HEPES, adjusted to pH 7.4. Background fluorescence was subtracted before calculating the ratio of F340/F380 nm to quantify intracellular calcium. Results are reported as mean ± SEM. Experiments were not randomly assigned; however, the experimenter was blinded to the experimental group and purpose.

### 2.5. Phagocytosis Assay

The phagocytosis assay was performed as described previously [47]. Briefly, RBL-2H3 wild-type (WT) and TRPV2 knockout (KO) cells were seeded at 1 × 10^4^ cells per well in flat-bottom translucent 96-well plates (TPP, Trasadingen, Switzerland) and cultured overnight in EMEM supplemented with 15% FBS at 37 °C and 5% CO_2_. The following day, the medium was replaced with EMEM containing 1% FBS, and cells were treated for 90 min with one of the following conditions: 10 nM phorbol myristate acetate (PMA) alone or 10 nM PMA with 300 μM Tranilast. After treatment, 10 μL of a sonicated pHrodoGreen-FBS mixture (1:1; Thermo Fisher Scientific, Waltham, MA, USA) diluted 1:10 in PBS was added to each well, and cells were incubated for 24 h at 37 °C and 5% CO_2_.

Following incubation, wells were washed three times with PBS, and images were acquired using an IX81 inverted fluorescence microscope (Olympus, Hamburg, Germany) equipped with a Hamamatsu Orca Flash 4 camera (Hamamatsu Photonics K.K., Hamamatsu City, Japan) at 40× magnification with a FITC filter. Fluorescence of phagocytosed particles was quantified as the mean grey value for each cell and corrected for cell-specific background using ImageJ 1.54g software (NIH, Bethesda, MD, USA).

### 2.6. Statistical Analysis

Experiments were conducted on cells derived from independent culture preparations on different experimental days. Each experimental day used cells from a single culture, and multiple experimental days were performed using different cell cultures to account for variability between preparations. The number of cells analyzed (*n*) refers to individual cells in patch-clamp and calcium imaging data. For calcium imaging, the number of independent culture preparations (*N*) is reported where applicable and reflects the number of biologically independent experiments. The number of experiments (*n*) in the phagocytosis Assay refers to different experimental days. Experiments were not randomly assigned, but the experimenter was blinded to the experimental group and purpose. Data were first tested for normality using the Shapiro–Wilk test. Comparisons between two groups were performed using a two-tailed unpaired Student’s *t*-test if data were normally distributed, or a Mann–Whitney U test if normality was not met. For comparisons involving more than two groups, one-way ANOVA was applied for normally distributed data with Tukey’s multiple comparisons test as a post hoc test; and the Kruskal–Wallis test was used for non-normally distributed data with Dunn’s multiple comparisons test as a post hoc test. Statistical analysis was performed on independent values using GraphPad Prism 10.4.1 (GraphPad Software Inc., La Jolla, CA, USA). Significance was assumed for *p* < 0.05, and * denotes *p* < 0.05, ** denotes *p* < 0.01, and *** denotes *p* < 0.001. Results are presented as mean ± SEM.

### 2.7. Declaration of AI and AI-Assisted Technologies in the Writing Process

During the preparation of this work, the authors used ChatGPT/OpenAI GPT-5.2 and DeepL Write/DeepL Pro in order to improve language and readability. After using this tool/service, the author(s) reviewed and edited the content as needed and take full responsibility for the content of the publication.

## 3. Results

### 3.1. Tranilast Does Not Inhibit TRPV2-Mediated Currents in HEK 293 Cells

**Figure 1:**
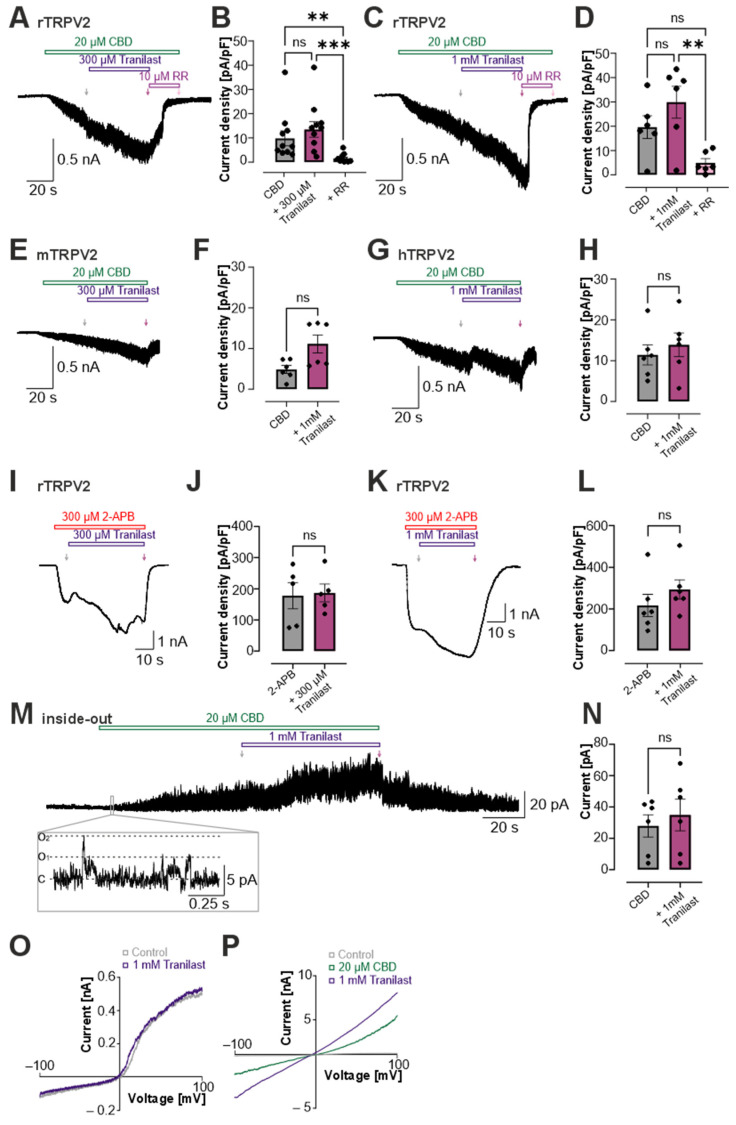
Tranilast does not directly inhibit TRPV2-mediated currents. (**A**,**C**) Whole-cell patch clamp recordings in HEK293 cells expressing TRPV2 showing activation of 20 µM CBD, which were not affected by co-application of tranilast at 300 µM or 1 mM and subsequent application of 10 µM ruthenium red (RR). (**B**,**D**) Bar diagrams showing current densities of membrane currents induced by CBD applied alone, CBD with 300 µM tranilast (**B**, *n* = 10; Kruskal–Wallis ANOVA with Dunn-Bonferroni post hoc test) or CBD with 1 mM tranilast (**D**, *n* = 6–10; one-way ANOVA with Tukey’s multiple comparisons test as post hoc test). Ruthenium red was applied at the end of the experiments. (**E**,**G**) Whole-cell patch clamp recording in HEK293t cells expressing mTRPV2 (**E**) and hTRPV2 (**G**) showing that currents induced by 20 µM CBD were not reduced by 1 mM tranilast. (**F**,**H**) Bar diagrams of mTRPV2 (**F**, *n* = 6, Mann–Whitney U test) and hTRPV2 (**H**, *n* = 6, Student’s *t*-test) showing current densities measured at the end of the application CBD alone or in with (**I**,**K**) Currents evoked by 300 µM 2-APB co-applied with 300 µM or 1 mM tranilast. (**J**,**L**) Bar diagram displaying the current densities of 2-APB-induced responses with 300 µM or 1 mM tranilast (n = 5–6; Student’s *t*-test). (**M**) Inside-out patch recordings displaying that 1 mM tranilast did not inhibit CBD-activated TRPV2 currents. The holding potential was set at +60 mV. The box represents a zoomed-in trace showing multi—channel openings induced by CBD. (To improve visualization of this trace, the low-pass digital filter function of the Fitmaster software was applied to smooth out high-frequency artifacts). (**N**) Bar diagram showing the current induced by CBD applied compared to the co-application with 1 mM tranilast (*n* = 6; Student’s *t*-test). (**O**,**P**) Membrane currents induced by 500 ms voltage-ramp protocols from −100 to +100 mV revealed no inhibition of basal (**O**) or CBD-induced (**P**) currents in the presence of 1 mM tranilast. In all experiments, current densities were quantified at the end of each agonist or inhibitor application, as indicated by arrows. ns denotes not significant, ** denotes *p* < 0.01, and *** denotes *p* < 0.001.

To assess whether tranilast directly inhibits TRPV2 channel activity, we performed whole-cell patch clamp recordings in HEK 293 cells stably expressing rat TRPV2. TRPV2 currents were first activated using the established agonists cannabidiol (CBD) and 2-aminoethyl diphenylborinate (2-APB) [48,49,50,51]. After channel activation, tranilast was co-applied with the agonist to test for potential direct inhibitory effects, thereby avoiding any misinterpretation due to washout phenomena. Although tranilast at concentrations below 100 µM has been used frequently in previous studies, we applied higher concentrations to minimize the risk of false-negative results [23,30,52]. Additionally, the short observation times inherent to patch-clamp experiments may require higher concentrations to reveal potential inhibitory effects. Application of 300 µM (Figure 1A, *n* = 11) or 1 mM tranilast (Figure 1C, *n* = 6) did not reduce rat TRPV2 (rTRPV2)-mediated currents evoked by 20 µM CBD. Rat TRPV2 remained fully active, with no inhibition or interruption of activation. Currents even often continued to increase, reflecting ongoing TRPV2 activity (Figure 1B,D). This does not indicate direct activation by tranilast. The subsequent application of the unspecific TRP channels blocker ruthenium red (RR, 10 µM) completely suppressed the currents, consistent with TRP-channel–mediated activity in TRPV2-expressing cells.

Because species-specific differences in TRPV2 pharmacology have been reported, we next examined whether the lack of inhibition might be restricted to rat TRPV2 [53]. We repeated the experiments using mouse TRPV2 (mTRPV2; Figure 1E, *n* = 6) and human TRPV2 (hTRPV2; Figure 1G, *n* = 6), applying CBD followed by co-application of 1 mM tranilast. We observed no inhibition in mTRPV2-expressing cells (Figure 1F) and only a very small, non-significant reduction in CBD-evoked currents in hTRPV2-expressing cells (Figure 1H). These findings indicate that tranilast does not substantially block TRPV2 in any of the tested species under our experimental conditions.

We next tested whether tranilast affects TRPV2-mediated currents activated by an alternative agonist. Membrane currents evoked by 300 µM 2-APB were similarly unaffected by co-application of 300 µM (Figure 1I, *n* = 5) or 1 mM tranilast (Figure 1K, *n* = 6). The corresponding bar diagrams showed no significant change at 300 µM (Figure 1J) or at 1 mM (Figure 1L) tranilast.

To rule out the possibility that a slow membrane permeability of tranilast may mask a rapid and direct inhibition through an intracellular mechanism, we also performed multi-channel inside-out patch recordings. Even under these conditions, activation by CBD was not significantly blocked by 1 mM tranilast, confirming that the compound does not directly inhibit the channel (Figure 1M,N, *n* = 6). In whole cell recordings, voltage ramps from −100 to +100 mV in the presence of 1 mM tranilast showed no detectable effects on basal currents (Figure 1O, *n* = 8), suggesting that tranilast does not directly activate TRPV2 or modify its voltage-dependent properties. In ramps where the TRPV2 was first activated by CBD, the co-application with tranilast did not reduce membrane currents (Figure 1P, *n* = 8).

### 3.2. Tranilast Does Not Inhibit Calcium Influx in Rat TRPV2-Expressing HEK293 Cells

**Figure 2:**
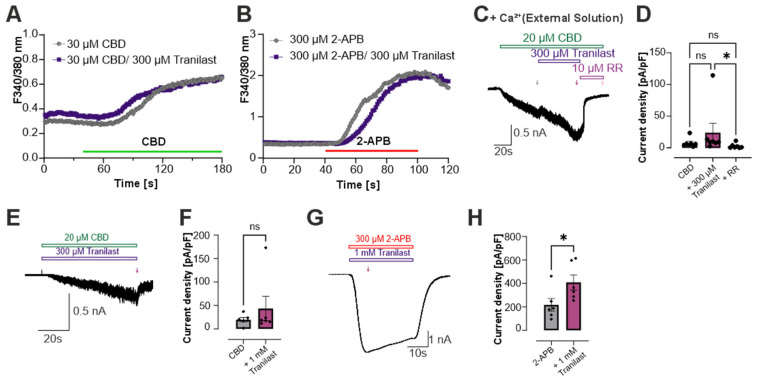
Tranilast does not inhibit calcium influx through TRPV2. (**A**) rTRPV2-expressing HEK293 cells were investigated by ratiometric calcium imaging. Either 30 µM CBD alone (grey curve, *n* = 798 cells, *N* = 2 preparations) or in combination with 300 µM tranilast (lila curve, *n* = 667 cells, *N* = 2 preparations) was applied. (**B**) Calcium imaging with either 2-APB alone (grey curve, 364, *N* = 2 preparations) or in combination with 300 µM tranilast (lila curve, *n* = 558, *N* = 2 preparations) was applied. In (**A**,**B**) the curves represent the mean Ca^2+^ response across all recorded cells, with mean ± SEM indicated. Data were collected across two independent experimental days. (**C**) Typical recording in HEK293 cells expressing rTRPV2, showing activation by 20 µM CBD in calcium-containing extracellular solution with co-application of tranilast and subsequently ruthenium red (RR). (**D**) Bar diagram comparing the current densities of membrane current induced by CBD applied alone or in combination with 300 µM tranilast or 10 µM RR (*n* = 7; Kruskal–Wallis ANOVA with Dunn-Bonferroni post hoc test). (**E**) rTRPV2-mediated currents induced by co-application of 1 mM tranilast and 20 µM CBD. (**F**) Bar diagram comparing the current density of CBD alone and co-application of 1 mM tranilast with CBD (*n* = 6, Mann–Whitney U test). (**G**) Co-application of 1 mM tranilast and 300 µM 2-APB. (**H**) Bar diagram comparing the current density of 2-APB alone and co-application of 1 mM tranilast with CBD (*n* = 6, Student’s *t*-test). In all experiments, current densities were quantified at the end of each agonist or inhibitor application, as indicated by arrows, except for Figure 2G, where the measurement was taken at the beginning of the 2-APB application to match the quantification time point used in Figure 1K. ns denotes not significant, * denotes *p* < 0.05.

Because previous studies have primarily reported on the inhibition of calcium entry by tranilast on TRPV2, we performed ratiometric calcium imaging in HEK293 cells stably expressing rat TRPV2 [16,17,23,27,30,41]. Application of 30 µM CBD elicited a robust and sustained increase in intracellular calcium concentration, consistent with TRPV2 activation. Co-application with 300 µM tranilast did not alter the amplitude or kinetics of this response (Figure 2A; CBD, *n* = 798; CBD + tranilast, *n* = 667).

When TRPV2 was activated with 300 µM 2-APB, the simultaneous application of 300 µM tranilast produced a brief delay in calcium rise but ultimately reached similar peak levels compared with 2-APB alone (Figure 2B; 2-APB, *n* = 364; 2-APB + tranilast, *n* = 558). To further assess whether extracellular calcium influences potential tranilast-mediated inhibition, we repeated patch clamp experiments on CBD-induced activation followed by co-application with 300 µM tranilast in calcium-containing extracellular solutions. However, no significant decrease or block was observed (Figure 2C,D; *n* = 7).

In addition, we performed whole-cell patch-clamp recordings in rTRPV2-expressing HEK293 cells with direct co- application of 1 mM tranilast to CBD (Figure 2E,F, *n* = 6) or 2-APB (Figure 2G,H, *n* = 6). Similar to the calcium imaging results, co-application of tranilast did not reduce TRPV2-mediated currents. The corresponding bar diagrams (Figure 2F,H) compare the inward currents evoked by 20 µM CBD or 300 µM 2-APB (as shown in Figure 1C,D,K,L) with those obtained during co-application of CBD and 1 mM tranilast (Figure 2E,F) or 2-APB + 1 mM tranilast (Figure 2G,H). CBD-evoked currents displayed only a small, non-significant change upon tranilast co-application, whereas 2-APB-evoked currents showed a significant increase in current density in the presence of tranilast, confirming that it does not inhibit TRPV2 activation by either agonist under these experimental conditions.

### 3.3. Tranilast Reduces Phagocytosis in RBL Cells

**Figure 3:**
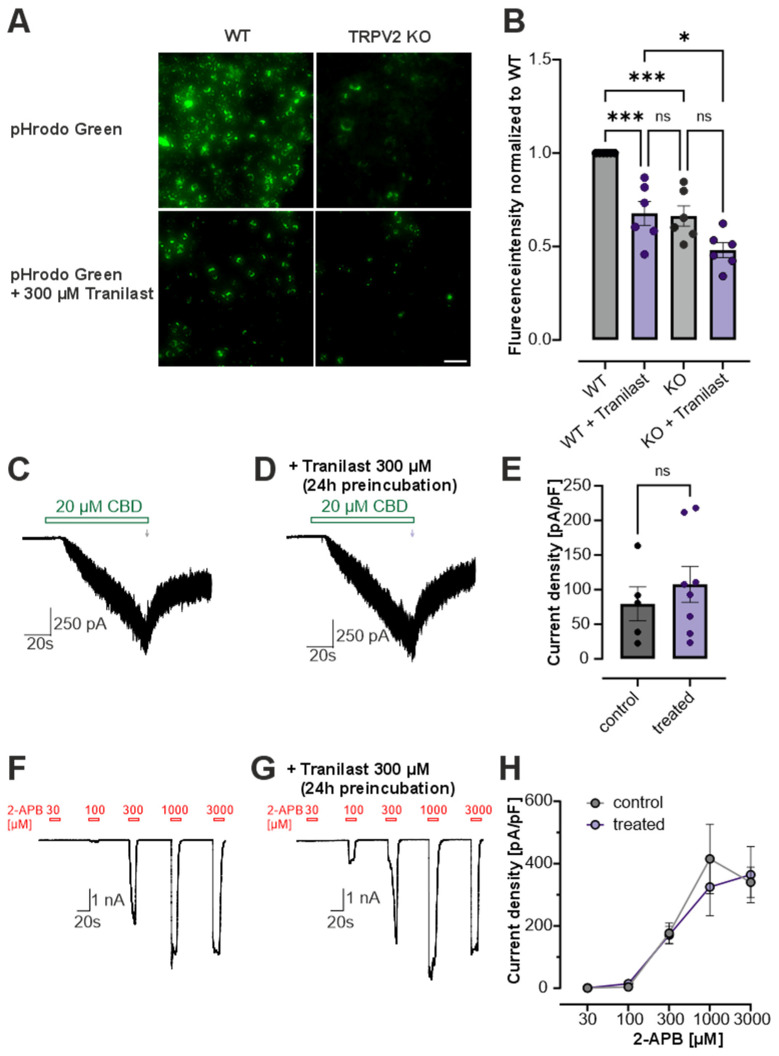
Tranilast reduces phagocytosis in RBL-cells. (**A**) Representative fluorescence images of pHrodo™ Green-labeled bioparticle uptake in wild-type (WT) and TRPV2 knockout (KO) RBL cells stimulated with PMA under control conditions or with 300 µM tranilast. Scale bar: 50 µm. (**B**) Quantification of phagocytic activity expressed as normalized fluorescence intensity relative to WT (*n* = 6; one-way ANOVA with Tukey’s multiple comparisons test as post hoc test. (**C**,**D**) Whole-cell patch clamp recordings in rTRPV2 expressing HEK293 cells displaying activation by 20 µM CBD alone (**C**) or after 24 h pre-incubation with 300 µM tranilast (**D**). Current density was quantified as indicated by arrows in the trace. (**E**) Bar diagram comparing the current densities of CBD-induced currents in control and tranilast-treated cells (*n* = 5–8; Student’s *t*-test). (**F**,**G**) Patch clamp traces showing 2-APB-induced concentration-dependent activation of control (**F**) and tranilast-treated cells (**G**). (**H**) Comparison of current densities between control cells (grey, *n* = 7) or cells after 24 h pre-incubation with 300 µM tranilast (lilac, *n* = 7); data are shown as mean ± S.E.M; ns denotes not significant, * denotes *p* < 0.05 and *** denotes *p* < 0.001.

To determine whether tranilast functionally reduces TRPV2-mediated effects at the cellular level, we next examined phagocytosis, known to be regulated by TRPV2 [2,27,47,54]. Earlier studies have reported that TRPV2 contributes to particle uptake in macrophages and that tranilast reduces this activity, presumably through TRPV2 blockade [27]. We used rat basophilic leukemia (RBL) cells in which we had previously established a CRISPR/Cas9-mediated TRPV2 knockout (KO) line [47]. In agreement with prior reports, wildtype RBL cells displayed a significantly higher level of phagocytosis compared to TRPV2-KO cells, confirming the involvement of TRPV2 in this process (Figure 3A,B, *n* = 6) [47]. Treatment of wild-type cells with 300 µM tranilast resulted in a marked reduction in phagocytic activity to levels comparable to those observed in TRPV2-KO cells (Figure 3A,B, *n* = 6). However, tranilast caused a modest, though not significant, decrease in phagocytosis in TRPV2-KO cells (Figure 3A,B, *n* = 6). These observations suggest that tranilast may indeed affect TRPV2-dependent processes such as phagocytosis. However, the partial reduction in phagocytic activity in TRPV2-deficient cells indicates that tranilast may also exert additional TRPV2-independent effects. Because our phagocytosis experiments involved prolonged tranilast exposure (24 h), we next investigated whether this long pre-incubation with tranilast affects TRPV2-mediated membrane currents in HEK293 cells. In cells incubated for 24 h with or without 300 µM tranilast, responses to 20 µM CBD as well as dose-dependent activation by 2-APB (30–3000 µM) were examined (Figure 3C–H, *n* = 5–8). Neither CBD-induced nor 2-APB-induced currents differed significantly between control and tranilast-treated cells, indicating that pre-incubation with tranilast does not impair TRPV2 channel activity (Figure 3E,H, *n* = 5–8).

### 3.4. Tranilast Reduces Oxidation-Induced Activation of TRPV2, TRPA1 and Voltage-Gated Na^+^ Channels

**Figure 4:**
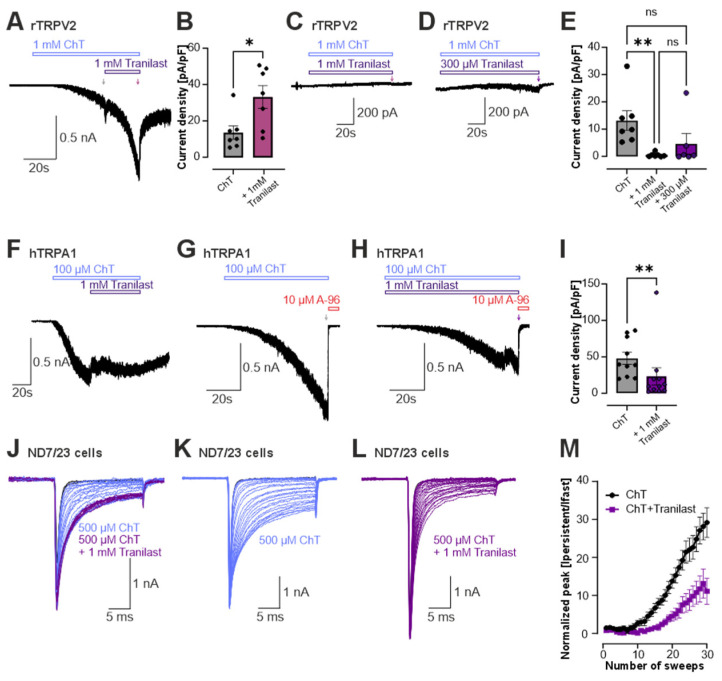
Tranilast reduces oxidation-induced activation of TRPV2. (**A**) Whole-cell patch clamp recordings in TRPV2-expressing HEK293 cells showing activation by 1 mM ChT, which was not affected by subsequent co-application of 1 mM tranilast. (**B**) Bar diagram showing the current density of ChT alone and co-applied 1 mM tranilast (*n* = 7; Mann–Whitney U test). (**C**,**D**) Current traces from rTRPV2-expressing cells following application of 1 mM ChT with co-application of 1 mM (**C**) or 300 µM (**D**) tranilast. (**E**) Bar diagram comparing the current density of ChT alone, co-application with 1 mM tranilast and 300 µM tranilast (*n* = 6–7; Kruskal–Wallis ANOVA with Dunn-Bonferroni post hoc test). (**F**) Typical recording showing hTRPA1 activation by 100 µM ChT in TRPA1-expressing HEK 293 cells, with subsequent co-application of 1 mM tranilast (*n* = 7). (**G**,**H**) Activation of hTRPA1 by 100 µM ChT (**G**) or co-application of 100 µM ChT and 1 mM tranilast (**H**). Currents were blocked with 10 µM of the TRPA1-blocker A967079 (A96). (**I**) Bar diagram comparing the current density of ChT applied alone or co-appliedwith 1 mM tranilast (*n* = 9–11; Mann–Whitney U test). (**J**–**L**) Representative current traces generated by voltage-gated Na^+^ channels endogenously expressed in ND7/23 cells. Cells were held at −120 mV and currents were evoked by thirty 20-ms-long pulses to 0 mV applied at 0.1 Hz. (**J**) Typical current traces from Na^+^ channels upon application of 500 µM ChT alone (blue) with subsequent co-application with 1 mM tranilast (lila) (*n* = 5). Typical current traces from Na^+^ channels upon application of 500 µM ChT alone (n = 9) (**K**), or co-application with 1 mM tranilast (*n* = 10) (**L**). (**M**) Development of normalized amplitudes of ChT-induced persistent currents over time in cells treated with either ChT alone (black) or ChT and tranilast (lila). For each trace, the amplitude of the persistent current was determined at 15 ms and then normalized to the peak amplitude of the transient fast current (Ipersistent/Ifast). ns denotes not significant, * denotes *p* < 0.05, ** denotes *p* < 0.01. In all experiments, current densities were quantified at the end of each agonist or inhibitor application, as indicated by arrows.

Tranilast has been reported to have antioxidant effects, and we previously demonstrated that TRPV2 is ROS-sensitive [54,55]. To examine whether tranilast interferes with oxidation-induced activation of TRPV2, we first activated TRPV2 with 1 mM chloramine T (ChT), an oxidizing agent known to activate TRPV2 [54]. Subsequent co-application with 1 mM tranilast did not reduce the current amplitude, indicating that tranilast does not reverse oxidation-induced TRPV2 activity once established (Figure 4A,B, *n* = 7). In contrast, the activation of TRPV2-mediated currents induced by ChT was strongly reduced when tranilast was co-applied throughout the experimental protocol (Figure 4C–E, *n* = 6–7). This indicates that tranilast can prevent the oxidation-mediated activation of the channel. Lower tranilast concentrations up to 300 µM also tended to suppress ChT-induced TRPV2 activation, but this effect did not reach statistical significance (Figure 4D,E, *n* = 6). To determine whether this effect reflects a general anti-oxidative property of tranilast, we next examined other ROS-sensitive ion channels. In HEK293 cells expressing human TRPA1, we first activated the channel with 100 µM ChT and subsequently co-applied 1 mM tranilast (Figure 4F, *n* = 7). Under these conditions, tranilast produced a slight current reduction, or at least prevented a further increase in ChT-induced currents (Figure 4F, *n* = 7). When ChT and tranilast were co-applied, channel activation was significantly decreased compared to ChT applied alone (Figure 4G–I, *n* = 9–10). Because voltage-gated Na^+^ channels are also well known to be highly sensitive to oxidative modification, we additionally tested whether tranilast affects ChT-induced oxidative activation of endogenous Na^+^ currents in ND7/23 cells [56]. In ND7/23 cells expressing voltage-gated Na^+^ channels, the application of 500 µM ChT induces large non-inactivating currents that persist throughout the 20 ms long test pulses applied at 0.1 Hz (Figure 4J, *n* = 5). When tranilast was added after the establishment of this ChT-induced removal of inactivation, no further increase in these persistent currents was observed (Figure 4J, *n* = 5). When 1 mM tranilast and 500 µM ChT were co-applied from the start of the protocol, the development of non-inactivating currents was strongly reduced (Figure 4J–M, *n* = 8). Of note, tranilast did not inhibit the fast transient peak amplitudes of Na^+^ currents. Thus, we were able to demonstrate a general anti-oxidative effect of tranilast on TRPV2, TRPA1 and Na^+^ channels.

## 4. Discussion

In this study, we examined whether tranilast acts as a direct inhibitor of TRPV2, a conclusion frequently drawn in earlier literature [23,24]. Using a combination of whole-cell and inside-out patch clamp recordings, calcium imaging and long-term incubation experiments, we demonstrate that tranilast does not inhibit TRPV2. Instead, our data support an alternative mechanism in which tranilast interferes with oxidation-dependent signaling, thereby modulating cellular processes in functional assays in which TRPV2, but also further ROS-sensitive mechanisms, participate.

The apparent discrepancy between numerous reports that tranilast inhibits TRPV2 and our electrophysiological evidence demonstrating no direct channel inhibition highlights the importance of reassessing earlier studies and validation of prior findings [17,27,28,29,30,32,33,34,35,36,37,38,39,40,41,42].

Our finding that tranilast prevents oxidation-driven activation of TRPV2, but also TRPA1 and Na^+^ channels, aligns with previous literature [55,57,58]. Miyachi et al. assessed the antioxidant property of tranilast by showing that it is able to significantly reduce ROS levels by acting as a ROS-scavenger [55]. Furthermore, 100 µM tranilast was demonstrated to reduce the production of hydrogen peroxide in phorbol myristate acetate (PMA)-stimulated macrophages [59]. As was previously demonstrated in macrophages [2,26,27], we found that tranilast reduces phagocytosis in PMA-activated RBL-cells. A similar reduction in phagocytosis was observed in RBL-cells lacking TRPV2, but tranilast still seemed to reduce phagocytosis in TRPV2-KO cells. It is possible that tranilast prevents phagocytosis by reducing a PMA-induced accumulation of ROS in RBL-cells. TRPV2 is directly activated by ROS [54], and thus the established role of TRPV2 for phagocytosis may depend on this ROS-sensitivity. Along these lines, the reduction in phagocytosis seen in tranilast-treated TRPV2-KO cells might be explained by a reduced activation of TRPM2, which has also been reported to be both ROS-sensitive and relevant for phagocytosis [60,61]. TRPM2 has also been shown to be expressed in mast cells [62].

Tranilast has also been reported to inhibit prostaglandin D_2_ (PGD_2_) synthase, an enzyme whose deficiency has been associated with impaired macrophage phagocytosis [63,64]. This mechanism may further contribute to the reduced phagocytic activity observed in TRPV2-KO RBL-cells treated with tranilast. Moreover, phagocytosis of S. aureus is known to involve TLR2 and NLRP3 signaling pathways, with PGD_2_ acting as a downstream regulator of these cascades [64]. Beyond this, tranilast has been shown to modulate NLRP3 inflammasome activation by affecting NF-κB-dependent transcription of NLRP3 and pro–IL-1β, providing an additional mechanism through which tranilast could influence innate immune functions and phagocytic responses [65]. However, it is important to note that these studies were conducted primarily in macrophages, whereas our experiments employed mast cells (RBL line), which may differ in their signaling pathways with distinct sensitivities to tranilast.

While tranilast markedly reduced oxidation-induced activation of TRPV2, its effect on TRPA1 was less pronounced, even though the tranilast-to-chloramine T ratio was higher (1 mM tranilast: 100 µM ChT). Previous studies have shown that cinnamoyl anthranilate derivatives (CADs), the structural class to which tranilast belongs, can directly modulate TRPA1, displaying partial agonist, desensitizing, or mixed agonist–antagonist properties depending on their substituents and concentrations [66]. In particular, the parent CAD compound exhibits moderate agonistic activity on TRPA1, while halogenated and electron-withdrawing variants produce stronger activation followed by desensitization at higher doses [66].

Although we never observed any tranilast-induced membrane currents in TRPA1-expressing cells, tranilast may itself act as a modulator of TRPA1 capable of inducing partial desensitization. Nevertheless, it seems likely that the reduction in chloramine T-induced TRPA1-activity by tranilast is mainly due to an anti-oxidative effect. This notion is supported by the ability of tranilast to reduce chloramine T-induced persistent Na^+^ currents in ND7/23 cells. Similar to TRPV2 [54], the ROS-sensitivity of Na^+^ channels that accounts for the development of these persistent currents is mediated by an oxidation of intracellular methionine residues [67]. Our data demonstrate that tranilast only prevents the development of persistent currents, but it fails to directly block both the persistent and the rapid peak currents. In our hands, these data strongly suggest that tranilast inhibits ROS-mediated effects without directly modulating Na^+^ channels.

In addition to basic research, there are clinical trials with tranilast based on the assumption that it blocks TRPV2 [34,35,36,38,39,40]. The beneficial actions of tranilast in some trials might derive from redox modulation rather than from a specific TRPV2-antagonism. Therefore, attributing the clinical efficacy of tranilast to TRPV2 may be misleading and could confound target validation efforts. Conversely, the anti-oxidative properties themselves may represent a useful therapeutic axis; clarifying which activity underlies specific clinical outcomes will aid in rational drug development.

## 5. Conclusions

Taken together, our work establishes that tranilast does not directly inhibit TRPV2 through blockade of the channel. Instead, tranilast modulates redox-dependent cellular processes by preventing oxidative activation of TRPV2 and other ion channels. These findings clarify the mechanism underlying prior observations and underscore the need for more specific tools to interrogate TRPV2 function in vitro and in vivo.

## Data Availability

All data generated during the current study are available from the corresponding author upon reasonable request.

## Abbreviations

The following abbreviations are used in this manuscript:

2-APB	2-Aminoethoxydiphenyl borat
CADs	cinnamoyl anthranilate derivatives
CBD	Cannabidiol
CHO	Chinese hamster ovary
ChT	Chloramin T
HEK293	human embryonic kidney
IGF-1	insulin-like growth factor 1
KO	knockout
NASH	non-alcoholic steatohepatitis
PDGF	platelet-derived growth factor
PMA	phorbol myristate acetate
PGD_2_	prostaglandin D_2_
RBL	rat basophilic leukemia
ROS	reactive oxygen species
RR	Ruthenium Red
TRP	Transient receptor potential
TRPA1	Transient receptor potential ankyrin 1
TRPV1	Transient receptor potential vanilloid 1
TRPV2	Transient receptor potential vanilloid 2
WT	wildtype
